# Alterations in muscular control when performing unfamiliar elbow flexion and extension movements

**DOI:** 10.1007/s00421-025-05791-5

**Published:** 2025-04-26

**Authors:** Elisa Romero Avila, Catherine Disselhorst-Klug

**Affiliations:** Department of Rehabilitation and Prevention Engineering, Institute of Applied Medical Engineering, RWTH Aachen University, Pauwelsstr. 20, 52074 Aachen, Germany

**Keywords:** Motor control, Muscle synergies, Muscular coactivation, Surface electromyography, Neuromechanics, Msotor learning

## Abstract

**Purpose:**

This work analyzes how the Central Nervous System (CNS) adapts its control strategies—muscle synergies and muscular coactivation—during unfamiliar elbow flexion/extension tasks at different velocities.

**Methods:**

Twenty healthy participants (10 male; 10 female; age 31 ± 10,2 years) were recruited. Muscular activation of the biceps brachii, brachioradialis, and triceps brachii was recorded using surface electromyography. Elbow movements were tracked using a motion-capture system and an upper body biomechanical model. To represent an unfamiliar task, participants performed the movement in the transverse plane, while the familiar task was performed in the sagittal plane to allow for comparison. Movements were executed at different angular velocities to assess their effect. Muscle synergies were identified using the Non-Negative Matrix Factorization method.

**Results:**

The results indicate that the CNS adapts to unfamiliar movements primarily by increasing muscular coactivation to control position and movement velocity (*p* < 0.001, comparing familiar versus unfamiliar tasks). In contrast, during familiar tasks, the CNS achieves the stability required for faster movements through a higher contribution of muscle synergies (*p* < 0.05, comparing slowest versus fastest velocity). The statistical results revealed no significant interaction between task familiarity and movement velocity, suggesting that the effect of task familiarity on muscular activation remains consistent across all angular velocities.

**Conclusion:**

This work provides valuable insights into how muscle synergies and muscular coactivation complement each other. For an unfamiliar elbow flexion/extension task, the CNS primarily adapts by increasing the activation of all muscles acting on the joint to control position and movement velocity.

**Supplementary Information:**

The online version contains supplementary material available at 10.1007/s00421-025-05791-5.

## Introduction

The central nervous system (CNS) employs different control strategies to coordinate and regulate movement, acquire new motor skills, and maintain joint stability. The supraspinal areas and the cortex are responsible for planning movements and coordinating the interaction with the environment, while the spinal cord regulates muscle activation through functional modules (Zych et al. 2021, 2018; Bizzi et al. 2008; Ivanenko et al. 2005; Bizzi and Cheung 2013; d'Avella and Bizzi 2005). These functional modules serve as units within the spinal cord that generate a specific motor response by exciting several muscles according to a given spatial and temporal structure (Bizzi et al. 2008; Grillner 1985; Ivanenko et al. 2005). Still, the CNS control must adapt to biomechanical constraints and environmental factors (Bizzi et al. 2008; Piek 2002; Thelen and Cooke 1987). While functional modules support motor control, they are not always the optimal solution; in some circumstances, the CNS may directly recruit individual muscles, particularly if no trained recruitment strategies of functional modules exist (Spencer and Thelen 2000). Moreover, in such cases, the CNS would utilize the redundancy of the muscular system to ensure movement precision. Here, redundancy of the neuromusculoskeletal system refers to conditions where muscles outnumber joint degrees of freedom (Bernstein 1967). However, debate remains on the contribution of the redundancy of the neuromusculoskeletal system and functional modules to control limb position, movement velocity, and the influence of experience on the control of such movements.

It is assumed that the functional modules help reduce the vast solution space for movement execution resulting from neuromusculoskeletal redundancy. These functional modules provide temporal and spatial input to the motor neurons, leading to the idea of muscle synergies (d’Avella and Bizzi 2005; d’Avella and Tresch 2002). Muscle synergies are part of the CNS’s “minimal intervention strategy” which refers to the control of only the elements that affect the task. Instead of activating single muscles, the CNS employs a modular approach to simplify movement control (Coscia et al. 2014; Bernstein 1967; Bizzi et al. 2008; Grillner 1985; Arbib 1981; Sohn and Ting 2016). Supraspinal areas regulate muscle activation timing through environmental sensory feedback, while spinal sensorimotor networks define the modules (Cheung et al. 2012).

Infant motor development suggests that functional modules and, thus, muscle synergies are not fully developed at birth, but rather arise with maturation, movement variability, and practice (Dewolf et al. 2021; Teulier et al. 2012). Consequently, the CNS appears to rely on activation of individual muscles, while taking advantage of the redundancy of the muscular system during spontaneous neonatal movements (Spencer and Thelen 2000). This results in a high degree of simultaneous activation of antagonistic and synergistic muscles (muscular coactivation), which represents a biomechanically inefficient pattern.

These findings support the hypothesis that unfamiliar movement tasks (either not well practiced or involving new external conditions) may initially manifest as muscular coactivation rather than in a modification of muscle synergies. Indeed, the CNS employs the simultaneous activation of agonist and antagonist pairs of muscles for maintaining joint stability and posture control, particularly while learning a new task (Moore et al. 2014). A study by Becker et al. showed that, during the presence of unknown environmental forces, muscular coactivation is used to control joint position (Becker et al. 2019). However, it has also been reported that, since muscular coactivation results in a high stiffness of the joint, an increased coactivation may impair motor control and movement coordination (Latash 2018; Nagai et al. 2011).

Examining elbow joint muscles in the context of control strategies is particularly interesting, since, according to Oosterwijk et al., the elbow joint significantly contributes to several Activities of Daily Living (ADL) and maintaining full elbow range of motion (ROM) is essential for carrying out these properly (Oosterwijk et al. 2018). In addition, muscular redundancy is lower in the elbow joint compared to other joints, which reduces the need for and the number of functional modules to simplify movement control. In this context, an effective control strategy for elbow movement is crucial for functionality in everyday life.

Von Werder et al. studied the contribution of the biceps brachii, brachioradialis, and triceps brachii in elbow flexion and extension movements at different velocities (von Werder and Disselhorst-Klug 2016). They found that, during concentric contractions, the biceps brachii and brachioradialis work as synergists with increasing muscle activation as the angular velocity increases. In eccentric contractions, the biceps brachii activation decreases, while the activation of the brachioradialis increases with increasing angular velocity. In addition, the activation of the triceps brachii muscle is increased when moving faster in both types of contraction. Interestingly, the increased activation of the brachioradialis does not coincide with the force–velocity relationship, suggesting a strategy of the CNS to control joint position and movement velocity (von Werder and Disselhorst-Klug 2016).

Given that muscle synergies are associated with the coordinated generation of uniform movements by groups of muscles and that muscular coactivation contributes to joint stability and posture control, our primary objective is to investigate and analyze the muscular control strategies utilized by the CNS when performing an unfamiliar elbow flexion/extension task with different movement velocities. We aim to understand better how muscle synergies and muscular coactivation complement each other in conditions involving the performance of an unfamiliar task at different movement velocities. Since all relevant muscle groups for elbow flexion/extension must be considered, changes in the muscular activation of the biceps brachii, brachioradialis, and triceps brachii have been investigated. However, given the low muscular redundancy of the elbow joint, which limits the potential for simplifying the control strategy through functional modules, we hypothesize that the CNS tends to use a direct, independent control strategy when performing unfamiliar movement tasks, that will manifest itself as increased muscular coactivation. Furthermore, we assume that the movement velocity affects central nervous control in both familiar and unfamiliar movement situations.

## Materials and methods

To investigate the effect of unfamiliar tasks on muscle coordination, elbow flexion/extension movements were performed in two conditions:

1.Sagittal plane: representing a familiar movement that is performed in various daily activities, executed against gravity.

2.Transverse plane: representing an unfamiliar movement, rarely performed in daily activities, not executed against gravity due to the arm being supported on a tabletop.

Each trial consisted of eight repetitions of flexion and extension movements, in which each repetition was carried out with a different angular velocity between 20°/s and 140°/s. Movement velocity was randomized for each repetition by giving the participants real-time visual feedback on the expected pace for each repetition (von Werder and Disselhorst-Klug 2016). Trials were repeated twice, separated by a 1-min pause to avoid fatigue. The protocol was repeated for each condition in random order.

### Data collection and processing

Twenty healthy participants (10 male; 10 female; age 31 ± 10,2 years, height 172 ± 7.9, weight 73.2 ± 11.5 kg) were recruited at the Dept. of Rehabilitation and Prevention Engineering of the RWTH Aachen University, Germany. The sample size was determined using a power analysis in G*Power (Faul et al. 2009) for a repeated-measures ANOVA with a medium effect size (*f*) = 0.25, correlation among repeated measures (*r*) = 0.5, power of 80%, error probability (*α*) = 0.05, and 8 measurements. All participants were right-hand dominant, and exclusion criteria included upper limb movement disorders. Participants were asked to avoid vigorous exercise on the day before the measurement. The Human Ethics Committee of the RWTH Aachen University approved this study, and all participants gave their written informed consent before the assessment.

A conventional pulley machine (Speed Pulley 50 kg, Lojer, Finland) was used to achieve constant external torque over the full range of motion and between the two conditions. A low external load (1 kg) was considered in both conditions to analyze the muscle activation of the biceps brachii, brachioradialis and triceps brachii. Although no maximal voluntary contraction (MVC) was assessed, the classification of the external load as a minimum resistance is supported by the previous studies conducted on young healthy individuals (von Werder and Disselhorst-Klug 2016; Farup et al. 2015; Jakobi and Rice 2002).

**Fig. 1:**
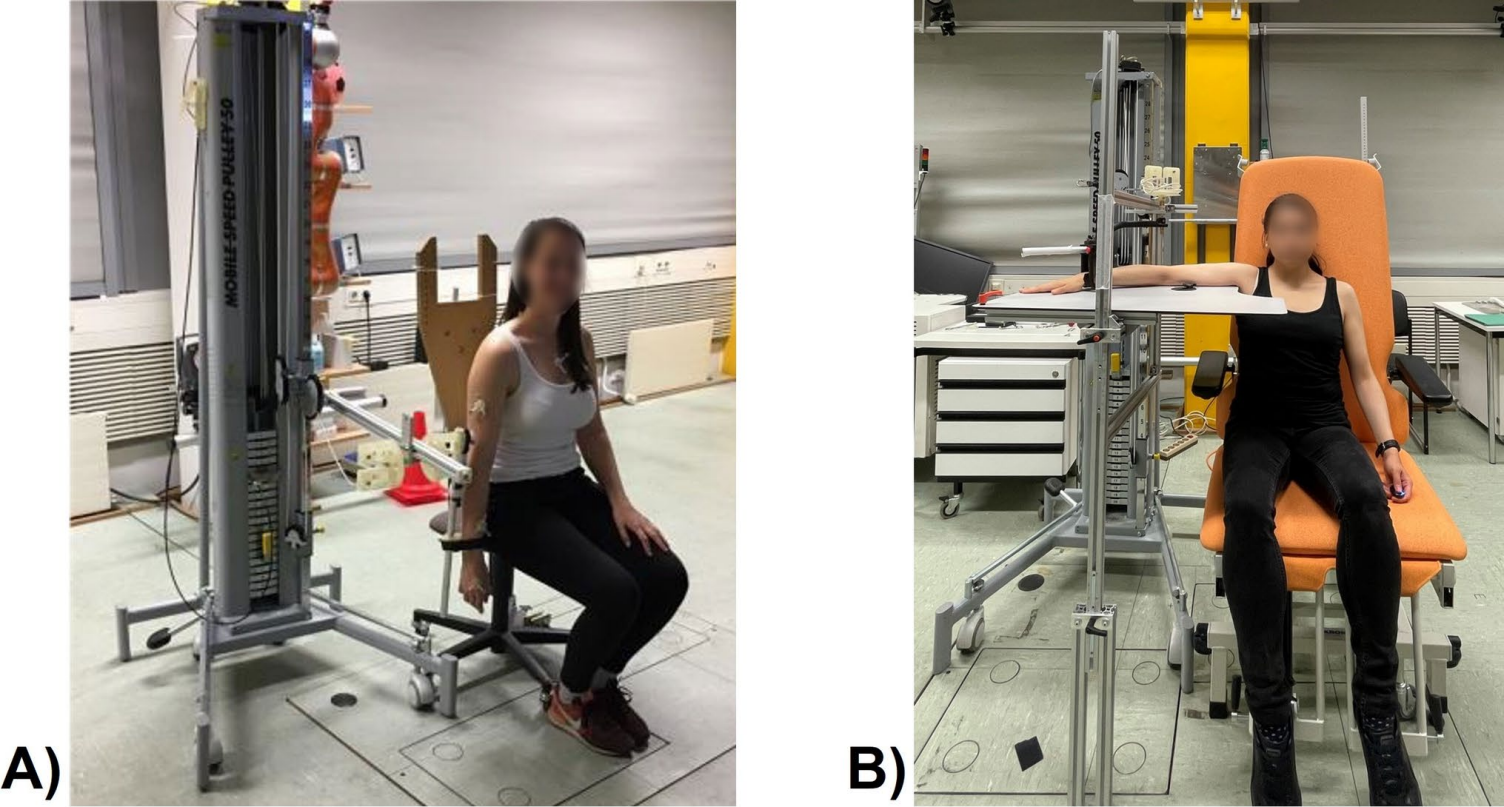
Setup of the pulley machine in (**a**) sagittal plane condition and (**b**) transverse plane condition

The participants were seated on a height-adjustable chair and fastened to the deflection pulley by the wrist, with the deflection pulley's midpoint aligning with the elbow flexion/extension axis. The deflection pulley generated a torque from the external load, which opposed elbow flexion movements (von Werder and Disselhorst-Klug 2016). In the sagittal plane condition, the shoulder joint was in a neutral position (Fig. 1a); in the transverse plane condition, the shoulder was in 90° abduction (Fig. 1b). A tabletop was included in the pulley machine to relieve the shoulder and avoid the execution of the movement against gravity (Pinter et al. 2010). Moreover, the tabletop minimized the effect of shoulder joint position on muscular activation changes.

The activation of the biceps brachii, brachioradialis, and triceps brachii muscles was recorded while performing the movement task with bipolar sEMG (Noraxon USA, Inc.; sampling frequency of 2000 Hz). This recording considered a gain of 1000, a bandpass filter covering from 1 to 500 Hz, and a 16-bit analog-to-digital converter. The preparation procedure included cleaning the participants’ skin with alcohol. Ag–AgCl pre-gelled electrodes (30 × 22 mm, Ambu Blue Sensor N, Ambu GmbH, Bad Nauheim, Germany) were placed with an inter-electrode distance of 20 mm. The placement and location of the electrodes for the biceps brachii and triceps brachii followed the SENIAM recommendations (Hermens et al. 2000). Based on the reference guidelines from Merlo et al., electrodes were placed around the muscle belly of the brachioradialis to minimize crosstalk while considering muscle anatomy and distance from the innervation zone (Merlo et al. 2021).

A 3D motion-capture system (Vicon Mx, Vicon Motion Systems Ltd UK, ten cameras, sampling rate of 200 Hz) was used to record the elbow’s kinematics. An upper body biomechanical model was used consisting of marker triads that were positioned in the thorax, upper and lower arm segments, and single markers for the shoulder, elbow, and wrist joint (Schmidt et al. 1999; Williams et al. 2006).

The kinematic data were processed to determine the elbow´s flexion/extension angles and angular velocities. The elbow angle signal was low pass filtered (Butterworth, 2nd order, fc 2.2 Hz), and the angular velocity was obtained through the gradient of this signal. The envelope of the sEMG signals was obtained through a sequence of signal processing procedures. These included a 9th-order Butterworth bandpass filter with a 10–500 Hz range, full-wave rectification, and smoothing via a moving average filter with a 500 ms window. Moreover, normalization followed the maximum mean method. For this, the maximum values of the signal were identified for each phase of the movement (either flexion or extension), for each muscle, and for each subject. These maximum values were then sorted, and the mean value of the top 50% of the highest maximum was calculated. This method is considered appropriate for comparing muscle synergies and activation patterns between different conditions (Besomi et al. 2020).

**Fig. 2:**
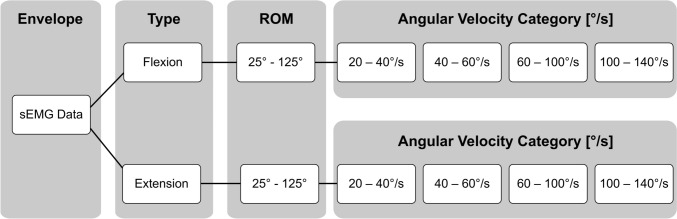
Categorization of the sEMG data according to the elbow’s range of motion (ROM), type of movement, and angular velocity

Following the kinematic and sEMG data processing, the sEMG envelopes were time-normalized and sorted into 8 categories using a modified decision tree algorithm detailed in Fig. 2 (Von Werder et al. 2015). This algorithm provides a structured examination of muscle activation during dynamic contractions by allowing the quantification and interpretation of the muscle activation patterns in quasi-isometric epochs (Avila et al. 2023). Based on the kinematic data, time-normalized sEMG sampling points were first categorized by the type of muscle contraction (concentric or eccentric), determined from the elbow flexion and extension angle signal. The angle range was set between 25° and 125°, and a second categorization identified whether the flexion/extension angle at each sampling point corresponded to this range. Mean angular velocity was calculated and sEMG envelope values were assigned to one of four angular velocity categories: 20–40°/s (0.34–0.69 rad/s), 40–60°/s (0.69–1.04 rad/s), 60–100°/s (1.04–1.74 rad/s), and 100–140°/s (1.74–2.44 rad/s). Finally, the root mean square (RMS) was computed from the sEMG envelope values within each category.

### Identification of the muscle synergies

The Non-Negative Matrix Factorization (NNMF) was considered to extract the number of muscle synergies (1–3) for each subject in both conditions within each angular velocity category and movement type. For each synergy identification, the NNMF algorithm was repeated 50 times with different initial random values, and the repetition with the highest coefficient of determination (*R*^2^), based on a threshold ≥ 85%, was considered for additional analysis (Coscia et al. 2014). The minimum number of muscle synergies was selected based on the requirement of whether it met or exceeded the specified threshold. The muscle synergy vector (W) representing the spatial output of the synergy was calculated. Temporal activation coefficients c(t) of the synergy were time-normalized and related to the elbow joint angle. Afterward, both conditions and each movement synergy activation coefficients c(t) and muscle synergy vectors (W) were averaged across all participants and across all velocity categories to investigate the effect of movement plane on both features. To assess the effect of movement velocity, muscle synergy vectors and activation coefficients were averaged across all participants of the lowest (20–40°/s) and highest (100–140°/s) movement category separately for each condition and each type of movement.

### Statistical analysis

For the statistical analysis, a two-way analysis of variance (ANOVA) was conducted using SPSS (IMB Corp. Version 29.0, NY). The significance level was set to *α* = 0.05. The independent variables (factors) were the movement conditions (familiar vs unfamiliar) and the angular velocity. ANOVAs were conducted to assess the main effects and interaction effects of these factors on elbow flexion, elbow extension, and each muscle. Additionally, Cohen’s d was calculated to measure effect size.

## Results

### Muscular activation patterns and coactivation

**Fig. 3:**
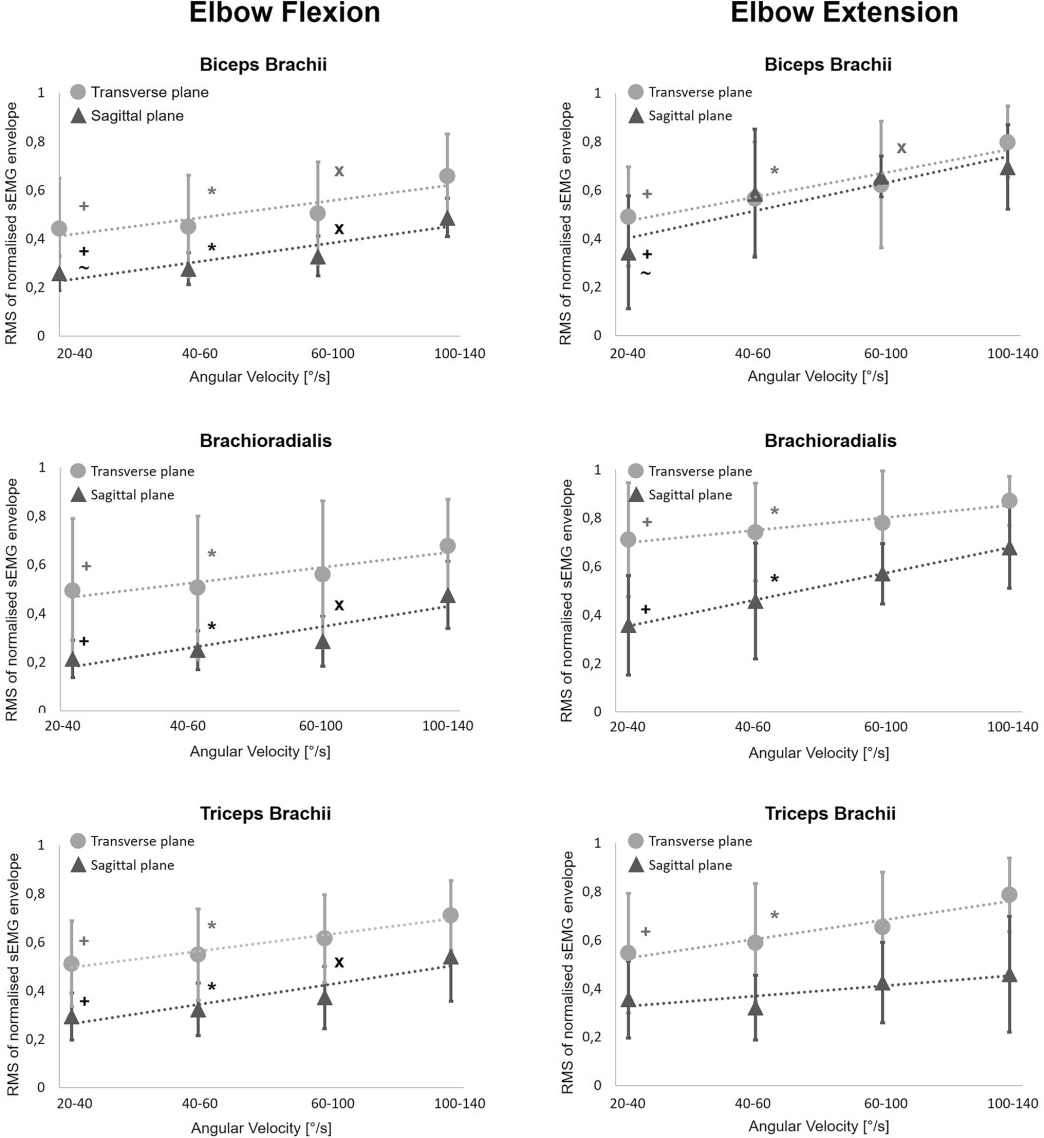
RMS of the sEMG envelope values in each movement velocity category recorded from biceps brachii, brachioradialis, and triceps brachii during elbow flexion and elbow extension. (▲) sagittal plane—familiar condition; (●) transverse plane—unfamiliar condition. Significance level *p* < 0.05: + between 20–40°/s and 100–140°/s; * between 40–60°/s and 100–140°/s; x between 60–100°/s and 100–140°/s; ~ between 20–40°/s and 60–100°/s

Figure 3 shows the RMS values of the sEMG envelopes for the three muscles while performing familiar and unfamiliar elbow flexion and extension movements. An increase in the RMS values of all three muscles with increasing angular velocity was observed during elbow flexion and extension in both conditions (Fig. 3 and Table 1). The movement velocity related increase in muscle activation during elbow flexion/extension movements was significant between slowest and the fastest velocity category in both conditions and in all muscles, except for the triceps brachii during elbow extension movements in the sagittal plane. Moreover, the effect size was large in all muscles and angular velocities (range: 0.704–1.904), except biceps brachii in the angular velocity categories 40–100°/s during elbow extension (Supplementary Table S1). No significant interaction between the angular velocity and sex was observed in both conditions.

**Table 1:** Mean RMS values of the biceps brachii, brachioradialis, and triceps brachii during different angular velocities and the two conditions: familiar (Fam.) and unfamiliar (Unf.). ANOVA results in relation to angular velocity categories and condition (familiar vs unfamiliar)

	Mean RMS values								ANOVA		
Angular velocity categories	20–40°/s		40–60°/s		60–100°/s		100–140°/s		*p* value velocity	*p* value condition	p-interaction
Condition	Fam	Unf	Fam	Unf	Fam	Unf	Fam	Unf			
**Elbow flexion**											
Biceps brachii	0.24	0.39	0.27	0.40	0.32	0.46	0.48	0.63	< 0.001*	< 0.001*	0.994
Brachioradialis	0.19	0.39	0.23	0.41	0.26	0.47	0.45	0.65	< 0.001*	< 0.001*	0.994
Triceps brachii	0.27	0.48	0.30	0.51	0.35	0.58	0.51	0.69	< 0.001*	< 0.001*	0.930
**Elbow extension**											
Biceps brachii	0.27	0.44	0.54	0.51	0.65	0.56	0.67	0.78	0.370	< 0.001*	0.238
Brachioradialis	0.30	0.67	0.40	0.71	0.55	0.75	0.65	0.86	< 0.001*	< 0.001*	0.469
Triceps brachii	0.32	0.49	0.29	0.53	0.39	0.61	0.39	0.77	< 0.001*	0.032*	0.402

**Fig. 4:**
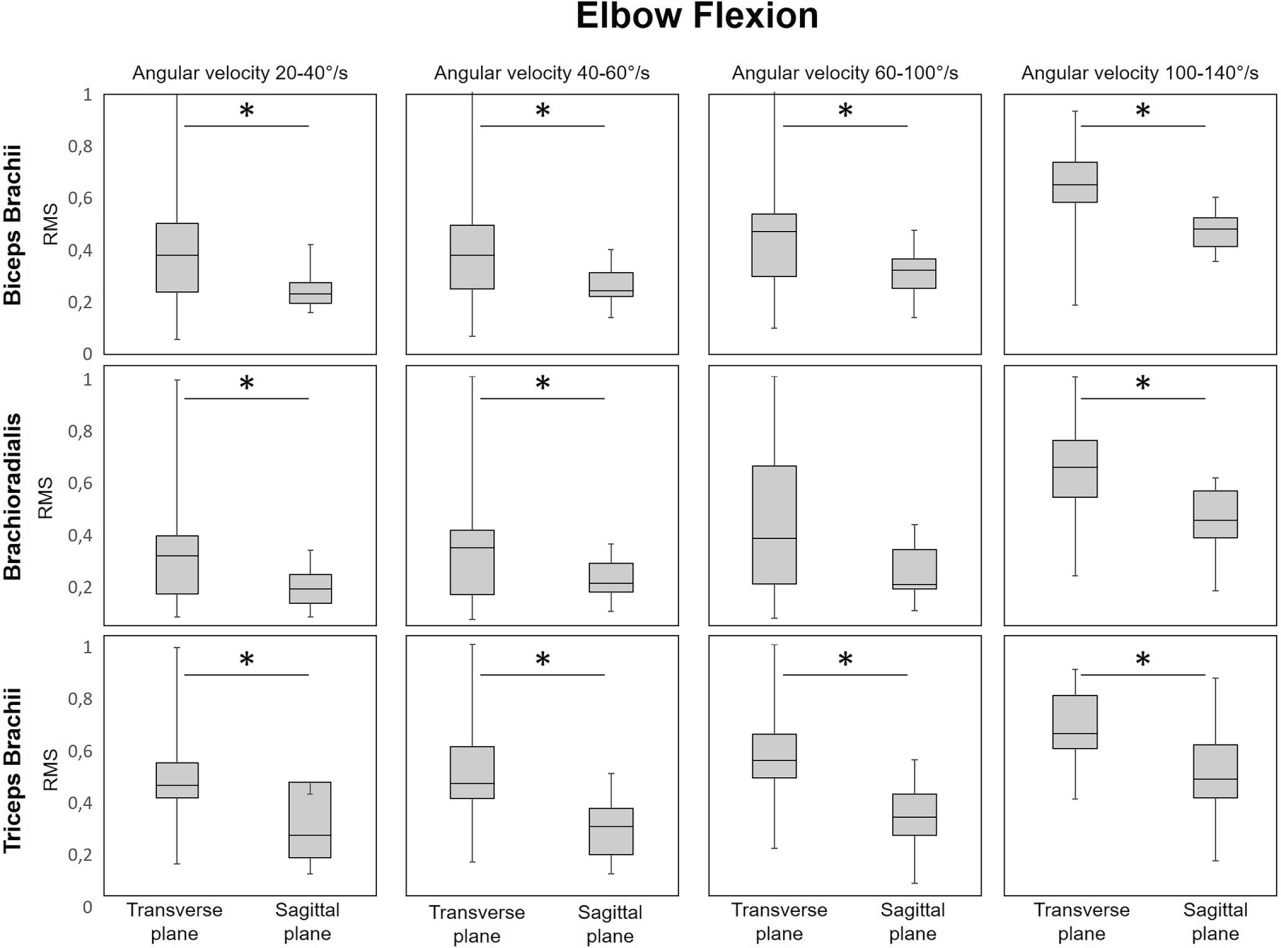
Comparison of the RMS values calculated from the sEMG envelope for biceps brachii, brachioradialis, and triceps brachii during elbow flexion: transverse (unfamiliar task) vs. sagittal (familiar task) plane across all angular velocity categories (*p < 0.01)

During elbow flexion, muscular coactivation increased significantly with increasing angular velocity in both planes (Fig. 4 and Table 1). However, the RMS values of all three muscles were significantly lower in the sagittal plane than during flexion movements in the transverse plane. Moreover, the ANOVA revealed no interaction effect between movement condition and angular velocity category (Table 1).

**Fig. 5:**
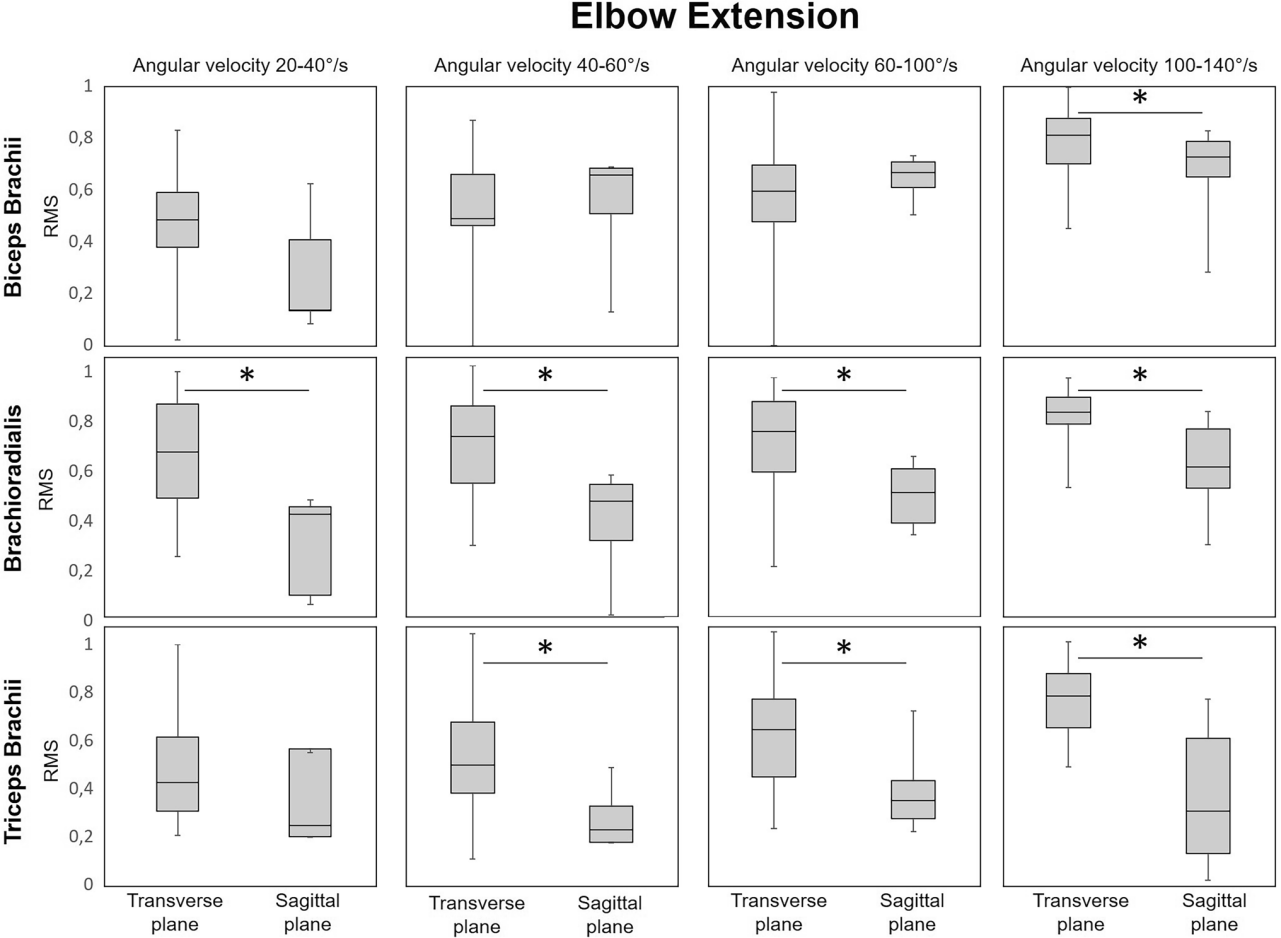
Comparison of the RMS values calculated from the sEMG envelope for biceps brachii, brachioradialis, and triceps brachii during elbow extension: transverse (unfamiliar task) vs. sagittal (familiar task) plane across the angular velocity categories. (*p < 0.01)

When the elbow was extended, the plane in which the movement was executed at different velocities affected the activation of each muscle differently (Fig. 5). At the highest angular velocity category (100–140°/s), the biceps brachii and triceps brachii activation during movements in the transverse plane was significantly higher compared to movements in the sagittal plane. Moreover, a significantly higher activation of the brachioradialis was observed when moving in the transverse plane compared to movements in the sagittal plane across all four angular velocity categories. As observed during elbow flexion, no significant interaction effect between movement condition and angular velocity category was detected in the ANOVA (Table 1).

### Muscle synergies

**Fig. 6:**
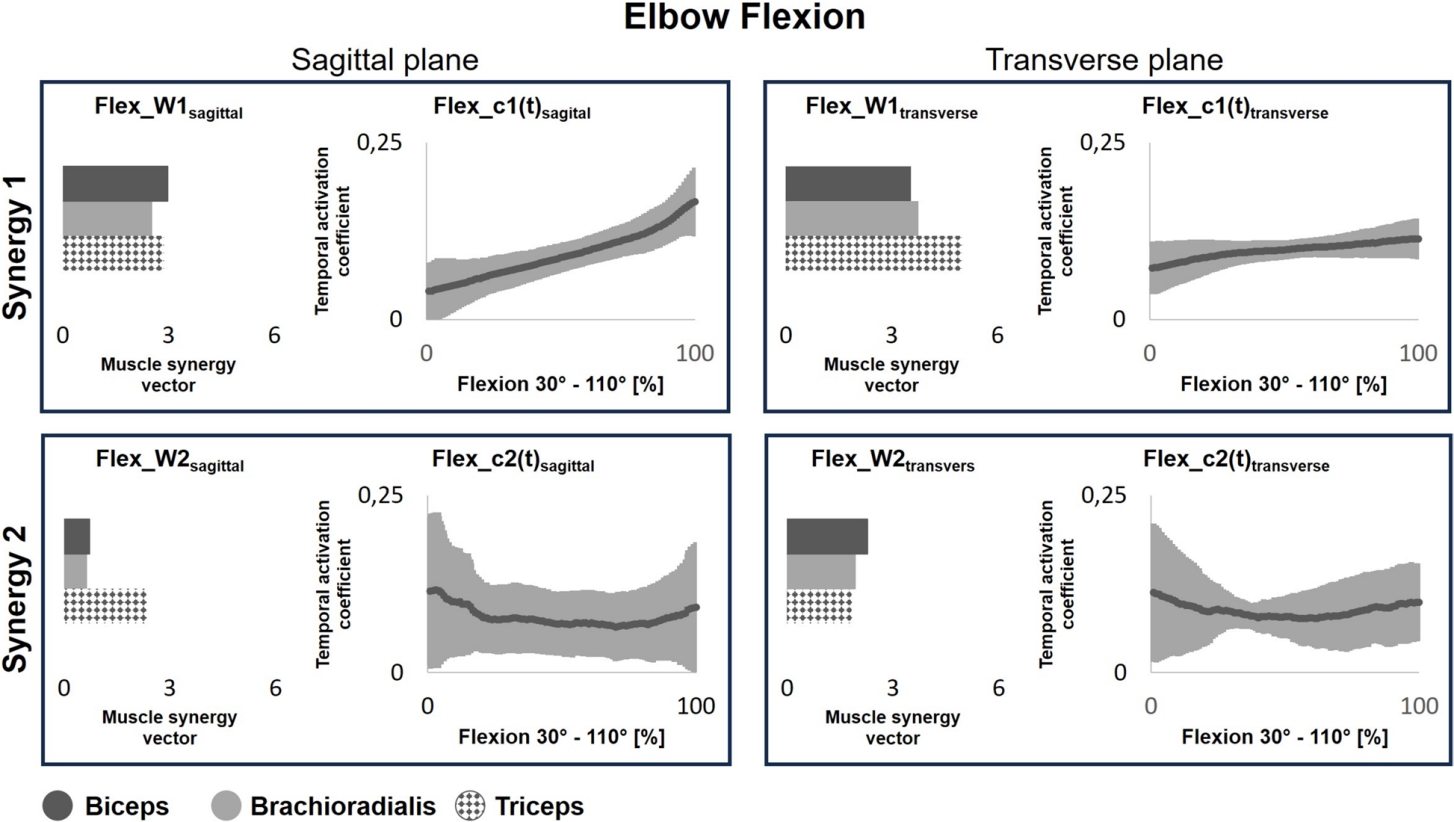
Muscle synergy temporal activation coefficients (c(t)) and muscle synergy vectors (W) during elbow flexion in the sagittal (familiar task) and transverse (unfamiliar task) plane. Greater c(t) represents a higher contribution of the muscle synergy to muscle activation, while W represents the weight of each muscle in the synergy. Features have been averaged across all participants and across all velocity categories

The number of muscle synergies did not differ significantly between flexion/extension movements in the sagittal and transverse planes (Supplementary Table S2). Figure 6 and Supplementary Tables S3 and S4 show the muscle synergy vectors and activation coefficients for elbow flexion in both planes. Synergy 1 activation coefficients (Flex_c1(t)_sagittal_) increased significantly in the sagittal plane compared to the transverse plane (Flex_c1(t)_transverse_). Moreover, synergy vector magnitudes were higher in the transverse plane (Flex_W1_transverse_), with biceps brachii contributing the most in the sagittal plane and triceps brachii in the transverse plane. An interaction effect between angular velocity and movement familiarity was only observed for the Brachioradialis in Synergy 1. Synergy 2 showed similar activation coefficients across conditions. Moreover, the spatial output was still related to the triceps brachii in the sagittal plane (Flex_W2_sagittal_), while in the transverse plane (Flex_W2_transverse_), output was evenly distributed among all muscles.

**Fig. 7:**
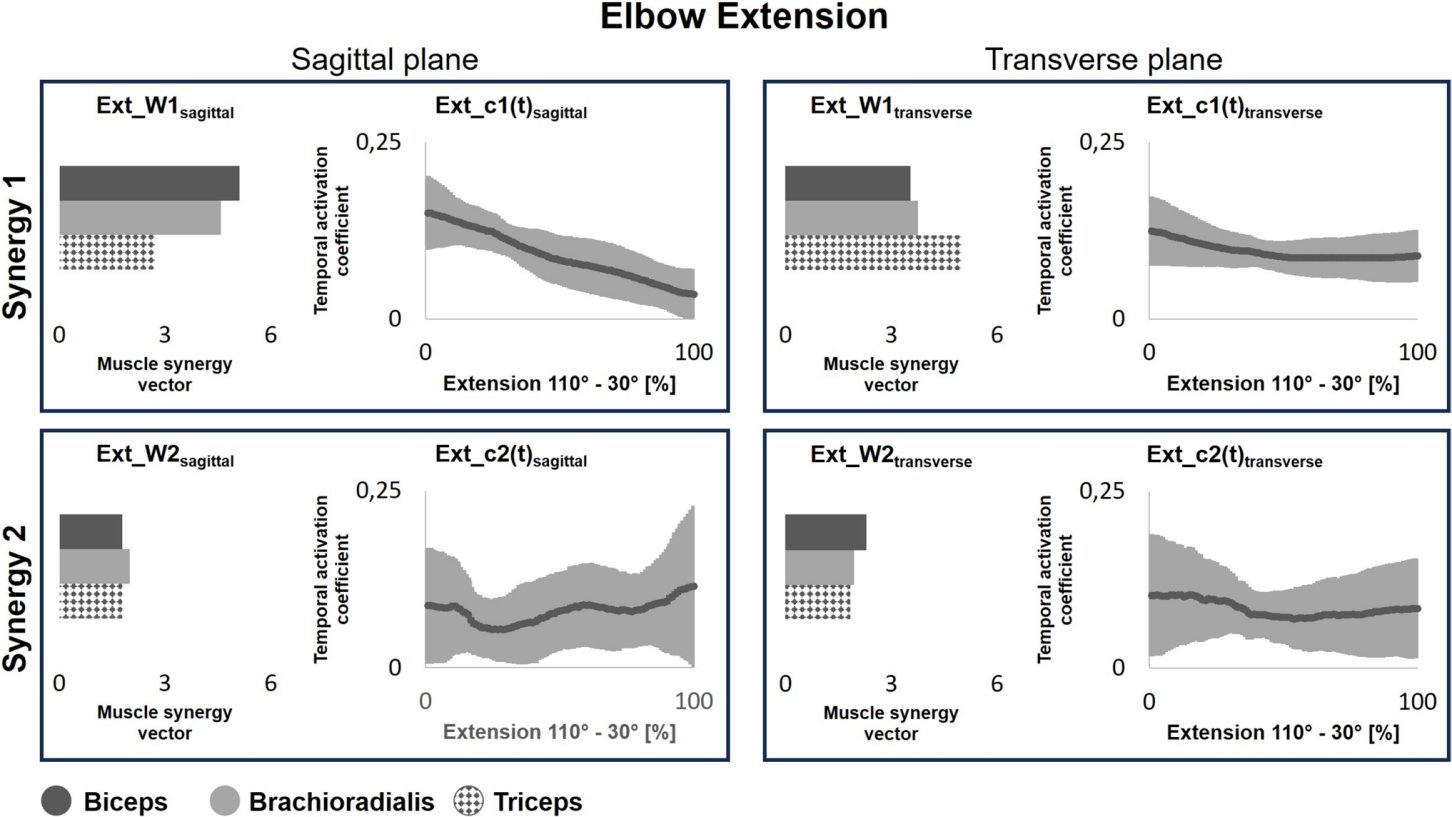
Temporal activation coefficients (c(t)) and muscle synergy vectors (W) during elbow extension in the sagittal (familiar task) and transverse (unfamiliar task) plane. Greater c(t) represents a higher contribution of the muscle synergy to muscle activation, while W represents the weight of each muscle in the synergy. Features have been averaged across all participants and across all velocity categories

Figure 7 and Supplementary Tables S3 and S4 show the muscle synergy vectors and temporal activation coefficients for elbow extension in the sagittal and transverse planes. Synergy 1 activation coefficients (Ext_c1(t)_sagittal_) decreased significantly in the sagittal plane. Additionally, Synergy 1 contributed most to biceps brachii activation in the sagittal plane (Ext_W1_sagittal_), while it contributed most to triceps brachii activation the transverse plane (Ext_W1_transverse_). No interaction effect between angular velocity and familiarity with the movement was observed in Synergy 1. Additionally, Synergy 2 showed no effects on the time component Ext_c2(t) of Synergy 2 or on the spatial output Ext_W2 during elbow extension.

**Fig. 8:**
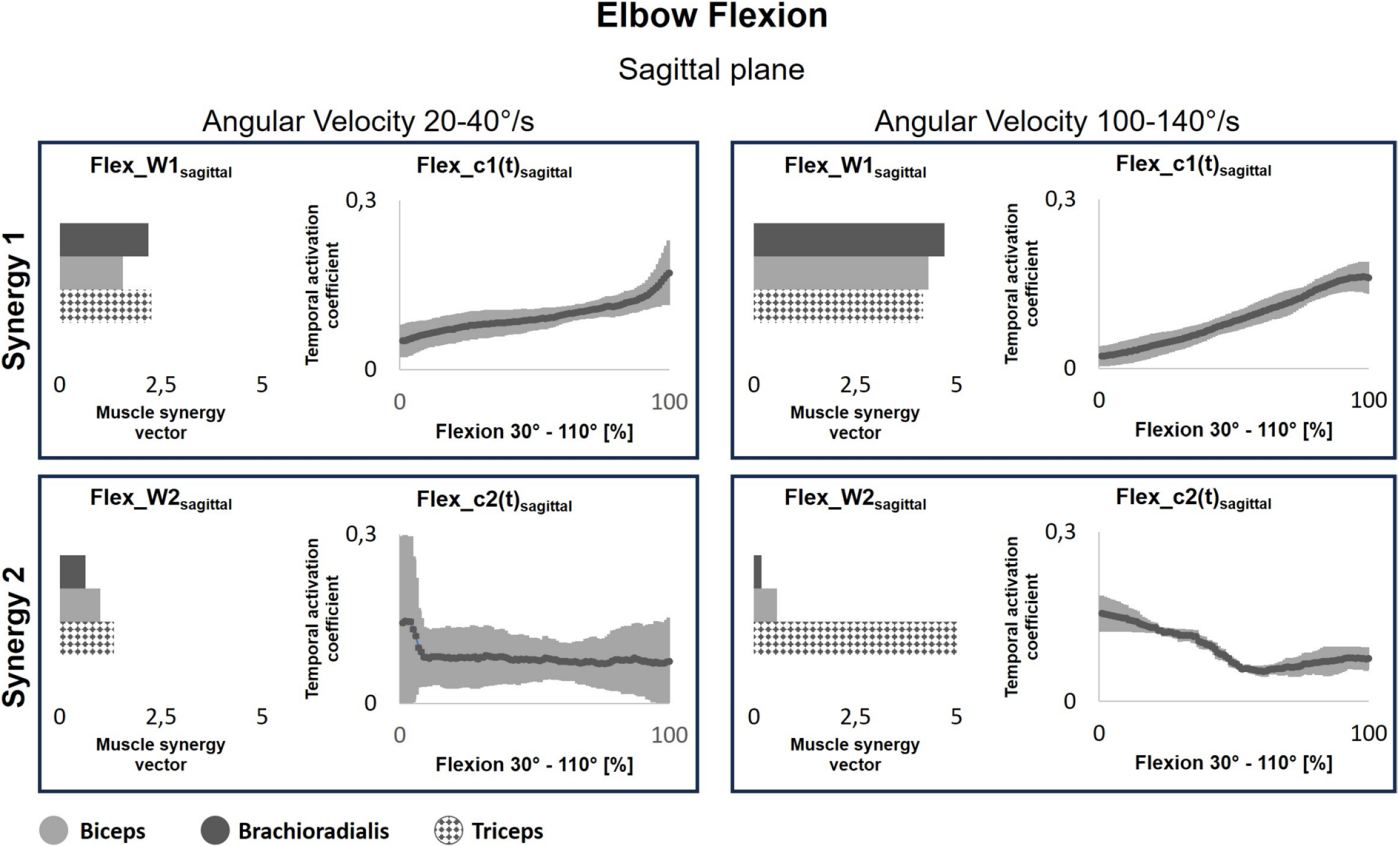
Temporal activation coefficients (c(t)) and muscle synergy vectors (w) during elbow flexion in the sagittal plane (familiar task). The left column displays the synergies at slower movement velocities (20–40°/s), and the right column shows the synergies when moving fast (100–140°/s). Greater c(t) represents a higher contribution of the muscle synergy to muscle activation, while W represents the weight of each muscle in the synergy

Figure 8 shows the muscle synergies during slow (20–40°/s) and fast (100–140°/s) elbow flexion in the sagittal plane. Not all participants were able to follow the real-time visual feedback accurately; as a result, no values could be computed for certain velocity categories. At the slowest angular velocity, Synergy 1 contributed equally to biceps and triceps brachii activation (Flex_W1_sagittal_), while at higher velocities, biceps brachii had the highest vector magnitude. Overall, the synergy vector magnitudes increased with movement velocity. Synergy 2 contributed most to triceps brachii activation regardless of movement velocity (Flex_W2_sagittal_). Moreover, its temporal activation coefficients (Flex_c2(t)_sagittal_) remained constant during slow movements but decreased with increasing elbow angle at higher speeds.

**Fig. 9:**
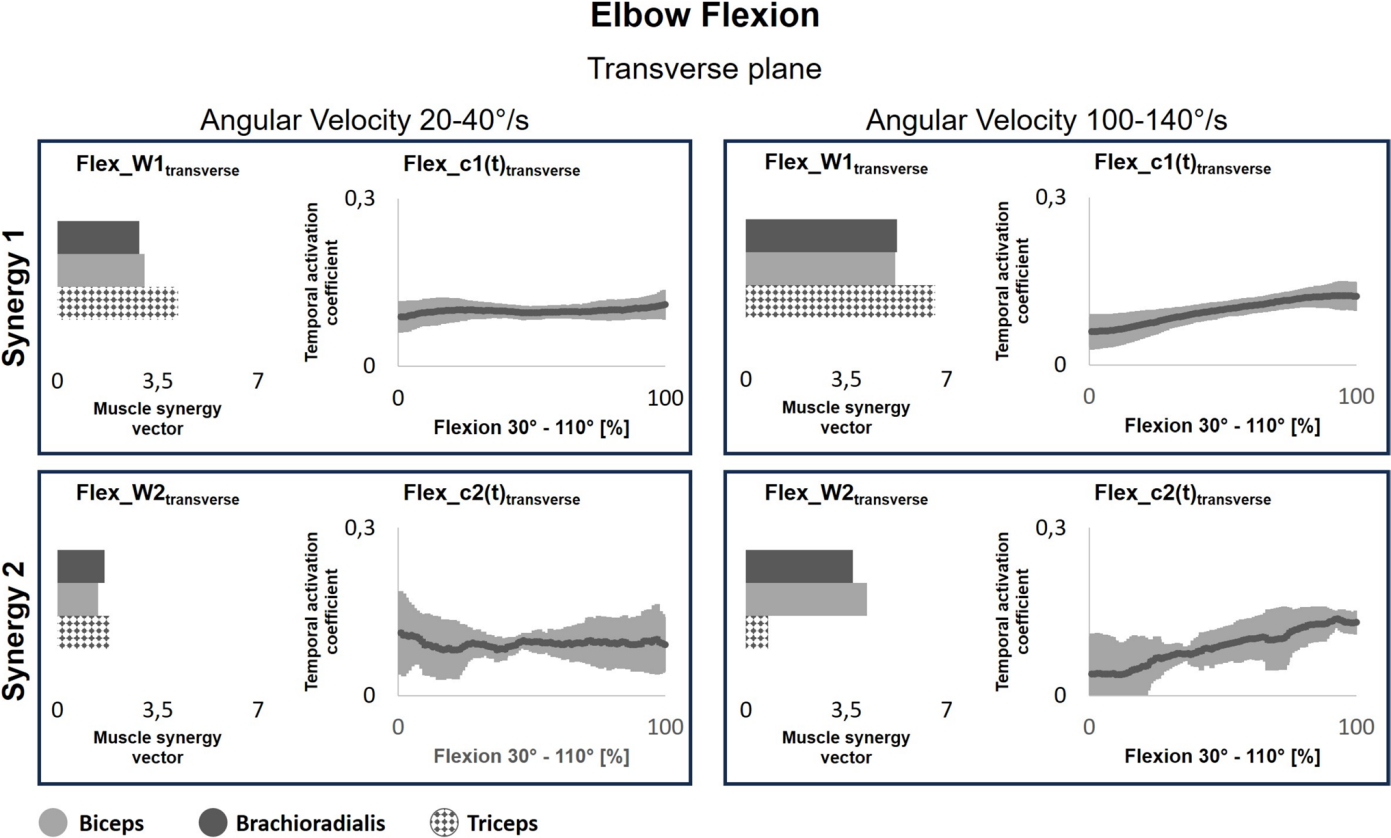
Temporal activation coefficients (c(t)) and muscle synergy vectors (W) during elbow flexion in the transverse plane (unfamiliar task). The left column displays the synergies when moving slower (20–40°/s), and the right column shows the synergies when moving fast (100–140°/s). Greater c(t) represents a higher contribution of the muscle synergy to muscle activation, while W represents the weight of each muscle in the synergy

During elbow flexion in the transverse plane, the magnitude of the muscle synergy vector (Flex_W1(t)_transverse_) was higher for all three muscles when moving faster (Fig. 9). Moreover Synergy 1 contributed most to triceps brachii activation temporal activation coefficients of Synergy 2 (Flex_c2(t)_transverse_) increased with faster velocities but remained constant during slow movements. Synergy 2 contributed more to the elbow flexor muscles at higher velocities (Flex_W1(t)_transverse_) but was evenly distributed among all muscles at slower velocities.

Regarding elbow extension, comparable results for the temporal activation coefficients and the muscle synergy vectors of Synergy 1 and Synergy 2 were also found in both conditions (Supplementary Figs. S1 and S2).

## Discussion

To our knowledge, this study is the first that examines muscle synergies and muscular coactivation while performing familiar and unfamiliar elbow flexion and extension tasks with different movement velocities. This issue has been addressed to better understand how these strategies complement each other, especially in conditions involving the performance of unfamiliar movement tasks executed in different movement velocities.

The results demonstrate that muscle activation increases with increasing movement velocity during both familiar and unfamiliar elbow flexion and extension movements. However, our results clearly demonstrate that the execution of less familiar movements is associated with increased muscular activation compared to the execution of well-experienced movements. The observed large effect size further supports the robustness of these results. The only exception is the biceps brachii muscle, where there is no significant difference in the level of muscle activation during elbow extension movements in both planes. However, assuming that the function of the biceps brachii is not so much the control of position and velocity but rather a weight-bearing function (von Werder and Disselhorst-Klug 2016), an effect of familiarity with the performed movement cannot be necessarily expected. On the other hand, it is worth noting that the consistently low RMS values for the triceps brachii during familiar elbow extension tasks across all angular velocity categories can be attributed to the fact that this movement was supported by gravity, requiring minimal triceps brachii activation.

The increase in muscular activation with increasing movement velocity remained consistent across all angular velocities between both conditions. This suggests that the CNS utilizes increased muscular activation to control movements at higher velocities, even during skilled movements like elbow flexion in the sagittal plane. Additionally, this increased activation contributed to the greater muscular coactivation observed in our study as angular velocity increased. According to Basmajian and De Luca, muscular coactivation involves the overactivity of all muscles responsible for the movement. Moreover, simultaneous activation of the agonist and antagonist muscles results in a reduced overall torque, as this torque represents the difference between the opposite forces of the agonist and antagonist muscles (Basmajian and De Luca 1985).

It is undisputed that the CNS activates agonist and antagonist muscle sets to achieve joint stability (Latash 2018; Suzuki et al. 2001; Frey-Law and Avin 2013). Due to the moment of inertia, it is also understandable that movements with higher velocities require a higher muscular activation to achieve the necessary joint stability. Thus, it can be expected that muscular coactivation increases with increasing movement velocity. Indeed, many groups have reported this. Suzuki et al. have investigated muscular coactivation during single-joint elbow movements in the transverse plane (Suzuki et al. 2001). They identified a positive correlation between maximum joint velocity and the amount of muscular coactivation remaining after movement execution and concluded that the CNS adjusts coactivation and, with that, joint stiffness in association with changes in movement velocity. This conclusion is supported by our data, which goes beyond the results of Suzuki et al. since it was possible to examine changes in muscular activation during the execution of the movement due to the categorization approach. Additionally, a larger range of velocities and different movement planes have been examined.

Regarding the muscle synergy analysis, no effect of movement velocity on the number of muscle synergies has been found. Additionally, no impact of velocity on the temporal activation coefficients of Synergy 1, which could be extracted in all participants, was detected during elbow flexion. Since activation coefficients represent how muscle synergies are modulated throughout the movement, and muscle synergy vectors indicate the contribution of each muscle to each synergy, these results suggest that the CNS utilizes the same functional modules when moving faster in skilled movement tasks. Nevertheless, comparing slow with fast movements, the contributions of Synergy 1 to the biceps and triceps brachii increase to the same extent when angular velocity increases. This means that the same temporal activation of the motor neuron pool is simply amplified when moving faster. Functionally, this amplification results again in a higher coactivation between the muscles. However, Synergy 2 does not indicate this effect of increasing coactivation with increasing velocity neither in familiar movements nor in unfamiliar movements. Since Synergy 2 could only be verified in a smaller proportion of the participants, it can be assumed that the estimation of Synergy 2 is less accurate and, consequently, that its statistical error is higher. This is even more evident at high movement velocities, which were not achieved by all participants.

Hug et al. have analyzed muscle synergies during pedaling across different torques and velocities in high-trained cyclists (Hug et al. 2011). They were able to show that independently from the mechanical constraints, the number of muscle synergies was fixed and that there was a robust consistency in the structure (muscle synergy vectors and temporal activation coefficients) of these synergies, particularly with respect to different torque–velocity combinations. The results of Hug et al. are consistent with those found in our study for movements in the sagittal plane, representing experienced movements. This is comprehensible, as only very experienced cyclists were included in the study by Hug et al. Both studies support the hypothesis that the CNS reacts to different mechanical movement constraints by modulating the muscle synergy recruitment (i.e., changes in muscle weightings) while keeping its structure constant (i.e., constant temporal activation coefficients) as long as familiar movements are performed. Furthermore, the results of our study indicate that when less familiar movements are performed, the CNS utilizes other controlling mechanisms, and the individual synergies become less relevant. In fact, the number, temporal activation coefficients, and muscle synergy vectors have been compared between experienced and unexperienced athletes, indicating an adaptation of intermuscular coordination with training (Santos et al. 2021; Barnamehei et al. 2018; Turpin et al. 2011; Kristiansen et al. 2015; Vaz et al. 2016; Taborri et al. 2018). Most studies show that the temporal activation of synergies does not differ between trained and untrained athletes (Vaz et al. 2016; Turpin et al. 2011; Barnamehei et al. 2018). However, differences between the groups have mostly been found in the muscle synergy vectors and their variability (Santos et al. 2021; Kristiansen et al. 2015; Turpin et al. 2011). The results of these studies are only partially consistent with the results of our study. Like the others, we also found a difference in the muscle synergy vectors between familiar and unfamiliar movements. In contrast to the other groups, we observed a change in the temporal activation coefficients. Torricelli et al. were able to show that neither spatially invariant nor temporally invariant muscle synergies alone were able to account for adaptions in muscle activation while experiencing new movement constraints (Torricelli et al. 2020). Furthermore, it seems that learning a new strategy within the same task implies some reorganization of the spatial structure in addition to a temporal adaption of existing underlying synergies. They have concluded that an adaptation in intermuscular coordination with movement experience manifests itself not only on the level of muscle synergies. This is in line with the results of our study, which indicate a higher coactivation in less familiar movements. Thus, increased activation of the agonist and antagonist muscles becomes the predominant form under the condition of unfamiliar movements. This not only influences the muscle synergy vector but also the temporal activation coefficients of the synergies, which now provide a smaller contribution to the movement (Saito et al. 2018).

The study has certainly limitations that should be considered when evaluating its conclusions. As mentioned in the results, not all participants were able to follow real-time visual feedback accurately during the kinematic data collection. This was not necessarily due to movement limitations, as none reported discomfort or pain during or after the measurements. Instead, the issue was likely related to the randomized velocity sequencing of the repetitions, which may have made it difficult for participants to track the feedback correctly. In the future, incorporating more repetitions should be considered to ensure that all angular velocity categories are properly represented in the data.

Another limitation was the absence of brachialis muscular activation recordings, as there is not enough evidence to reliably support its recording using sEMG (Staudenmann and Taube 2015). However, the analysis of the primary muscles involved in the movement (biceps brachii, brachioradialis, and triceps brachii), provided reliable insights into the control strategies for elbow flexion/extension movements. A key point is whether elbow flexion/extension movements are an appropriate model for investigating the targeted research objectives. However, these elbow flexion/extension movements are essential for performing many ADLs ranging from bathing a specific body area, brushing teeth, drinking from a glass, to pulling up socks. In addition to their relevance to everyday tasks, the advantage of these movements is their relative simplicity in biomechanical terms, which allows for comparable conditions across different conditions. In total, there are no more than four muscles acting via the elbow joint, and three of them are easily accessible for sEMG recordings, so that the muscle activation that induces and controls the movement can be recorded nearly in its entirety. This is important when talking about coordination and force sharing of different muscles. However, this advantage is at the same time a disadvantage, because if only the muscular activity of three muscles is known, in principle no more than three synergies can be determined by NNMF algorithms. Several strategies were implemented to compensate for the small set of muscles considered for the muscle synergy analysis. Besides evaluating the relevant muscles for elbow flexion and extension, the sEMG data were normalized to the maximum mean values for each movement phase and subject. This balanced the effect of electrode position and muscle characteristics, thus allowing a more reliable identification of muscle coordination patterns and changes in muscle synergies, even with a low number of muscles (Besomi et al. 2020). Finally, the sEMG envelopes were categorized according to type of contraction, ROM, and angular velocity. Based on the kinematic data, this categorization method provided a better understanding of changes in muscular coordination patterns according to these biomechanical characteristics.

It remains debatable whether basic movements such as elbow flexion/extension represent the complexity of the solution strategy employed by the CNS to solve familiar and unfamiliar movement tasks at different movement speeds. This brings us to the second point: What are familiar and unfamiliar movements? Elbow flexion in the sagittal plane is one of the most frequently performed movements in our daily lives. However, the deaccelerated extension of the elbow in the sagittal plane, as is used, for example, to place a cup on a table, and as it was examined in this study, occurs much less frequently. Much more often, elbow extension is assisted by gravity, allowing the arm to simply drop into an extended position. Consequently, the level of familiarity with a movement is already different for flexion and extension movements performed in the sagittal plane condition: Despite all this, the results of the study are largely unambiguous and, in many aspects, support current theories about neuromuscular control.

## Conclusion

This study analyzed differences in motor control during the execution of familiar and unfamiliar movements performed with different movement velocities. The analysis of changes in the structure of the muscle synergies and muscular coactivation confirmed our hypothesis that the CNS’s adaptive response focuses primarily on an increased activation strategy of all muscles acting on the joint to control position and movement velocity. During the performance of familiar movement tasks, such as elbow flexion, the CNS achieves the stability required for faster movements through a higher contribution of muscle synergies. For unfamiliar movement tasks, i.e., velocity-controlled elbow extension, the joint stability necessary for faster movement execution seems to be achieved by increased muscular coactivation. The results of the study contribute to a better understanding of how muscle synergies and muscular coactivation complement each other. However, the study also shows the complexity of motor control in different biomechanical conditions. Further research is needed on healthy participants and, if possible, on patients with neuromuscular disorders to get deeper insights into physiological and pathological muscular control.

## Supplementary Information

Below is the link to the electronic supplementary material.

Supplementary file1 (DOCX 484 KB)

## Data Availability

All data supporting the findings of this study are available on request from the corresponding author.

## Abbreviations

ADL: Activities of daily living
CNS: Central nervous system
DOF: Degrees of freedom
NNMF: Non-negative matrix factorization
RMS: Root mean square
ROM: Range of motion
R^2^: Coefficient of determination
sEMG: Surface electromyography
