# Supplementary material for: Alterations in muscular control when performing unfamiliar elbow flexion and extension movements
Source: Eur J Appl Physiol. 2025 Apr 26;125(10):2765–79. doi: 10.1007/s00421-025-05791-5 (PMC12479688; doi:10.1007/s00421-025-05791-5)
Supplement: Supplementary file 1 — Supplementary file1 (DOCX 484 KB) [file 421_2025_5791_MOESM1_ESM.docx]

Alterations in Muscular Control When Performing Unfamiliar Elbow Flexion and Extension Movements

**Authors: Elisa Romero Avila, Catherine Disselhorst-Klug**

**Affiliation:**

Dept. of Rehabilitation & Prevention Engineering, Institute of Applied Medical Engineering,
RWTH Aachen University. Pauwelsstr. 20 52074 Aachen, Germany.

**ORCiD:**

0000-0001-8292-4271 Elisa Romero Avila

0000-0001-7624-6961 Catherine Disselhorst-Klug

**Corresponding Author:**

Elisa Romero Avila

Email: [romero@ame.rwth-aachen.de](mailto:romero@ame.rwth-aachen.de)
Tel: +49 241 80-87345

# **Supplementary Information**

## **Additional results to the section “Muscular activation patterns and coactivation”**

**Table S1.** Effect sizes (Cohen’s d) for muscular activation differences between familiar and unfamiliar tasks in the biceps brachii, brachioradialis and triceps brachii during elbow flexion and extension. Effect size values and their corresponding descriptions are provided.

|  | **Effect size (Cohen’s D) and descriptions** | | | | | | | | |
| --- | --- | --- | --- | --- | --- | --- | --- | --- | --- |
| **Angular velocity categories** | *20 – 40 °/s* | | *40 – 60 °/s* | | *60 – 100 °/s* | | *100 – 140°/s* | | |
| **Elbow Flexion** |  |  |  |  |  |  |  |  |  |
| Biceps brachii | 0,896 | Large | 0,798 | Large | 0,849 | Large | 0,872 | Large |  |
| Brachioradialis | 0,847 | Large | 0,758 | Large | 0,855 | Large | 0,840 | Large |  |
| Triceps brachii | 1,353 | Large | 1,316 | Large | 1,475 | Large | 1,059 | Large |  |
| **Elbow Extension** |  |  |  |  |  |  |  |  |  |
| Biceps brachii | 0,836 | Large | -0,096 | Small | -0,356 | Small | 0,698 | Small |  |
| Brachioradialis | 1,594 | Large | 1,485 | Large | 0,964 | Large | 1,510 | Large |  |
| Triceps brachii | 0,704 | Large | 1,024 | Large | 1,010 | Large | 1,904 | Large |  |

## **Additional results to the section “Muscles synergies”**

**Statistical analysis on muscle synergies**

**Table S2.** Effect of angular velocity and condition (familiar and unfamiliar task) on the number of muscle synergies

| **Source** | *p-value* |
| --- | --- |
| **Familiar vs Unfamiliar task**  (FUT) | 0.960 |
| **Angular velocity categories**  (AVC) | 0.499 |
| **Interaction**  FUT*AVC | 0.734 |

**Table S3.** Effect of angular velocity and condition (familiar and unfamiliar task) on the muscle synergy vectors of Synergy 1. * indicates significant differences.

|  | **Mean** | | | | | | | | **ANOVA** | | |
| --- | --- | --- | --- | --- | --- | --- | --- | --- | --- | --- | --- |
| **Muscle synergy vectors** | *20 – 40 °/s* | | *40 – 60 °/s* | | *60 – 100 °/s* | | *100 – 140°/s* | | *p-value velocity* | *p-value condition* | *p- interaction* |
| **Condition** | *Fam.* | *Unf.* | *Fam.* | *Unf.* | *Fam.* | *Unf.* | *Fam.* | *Unf.* |  |  |  |
| **Elbow Flexion** |  |  |  |  |  |  |  |  |  |  |  |
| Biceps brachii | 1.59 | 2.58 | 1.76 | 1.58 | 2.14 | 2.73 | 5.03 | 3.66 | 0.025* | 0.993 | 0.539 |
| Brachioradialis | 1.04 | 2.84 | 1.59 | 3.36 | 2.12 | 3.33 | 5.69 | 2.42 | 0.156 | 0.517 | 0.031* |
| Triceps brachii | 2.09 | 3.39 | 2.28 | 3.59 | 2.07 | 3.64 | 2.58 | 7.38 | 0.022* | <0.001 | 0.092 |
| **Elbow Extension** |  |  |  |  |  |  |  |  |  |  |  |
| Biceps brachii | 3.52 | 3.95 | 5.07 | 3.16 | 5.92 | 4.40 | 5.44 | 5.78 | 0.507 | 0.538 | 0.752 |
| Brachioradialis | 2.24 | 4.52 | 2.92 | 4.84 | 3.16 | 5.25 | 5.46 | 6.72 | 0.058 | 0.041* | 0.963 |
| Triceps brachii | 0.12 | 3.46 | 0.41 | 3.96 | 0.98 | 4.44 | 2.65 | 6.02 | 0.072 | <0.001* | 0.998 |

**Table S4.** Effect of angular velocity and condition (familiar and unfamiliar task) on the temporal activation coefficients of Synergy 1. * indicates significant differences.

| **Temporal activation coefficients** | *p-value velocity* | *p-value condition* |
| --- | --- | --- |
| Elbow flexion | 0.227 | <0.001* |
| Elbow extension | 0.003* | 0.071 |

**Changes in muscle synergies during slow (20 - 40°/s) and fast (100 – 140°/s) elbow extension in the sagittal plane and transverse plane.**

Muscle synergy results of elbow extension in the sagittal (Fig. S1) and transverse (Fig. S2) planes. The movement in the sagittal plane represents a familiar task, while the movement in the transverse plane is the unfamiliar one.

**
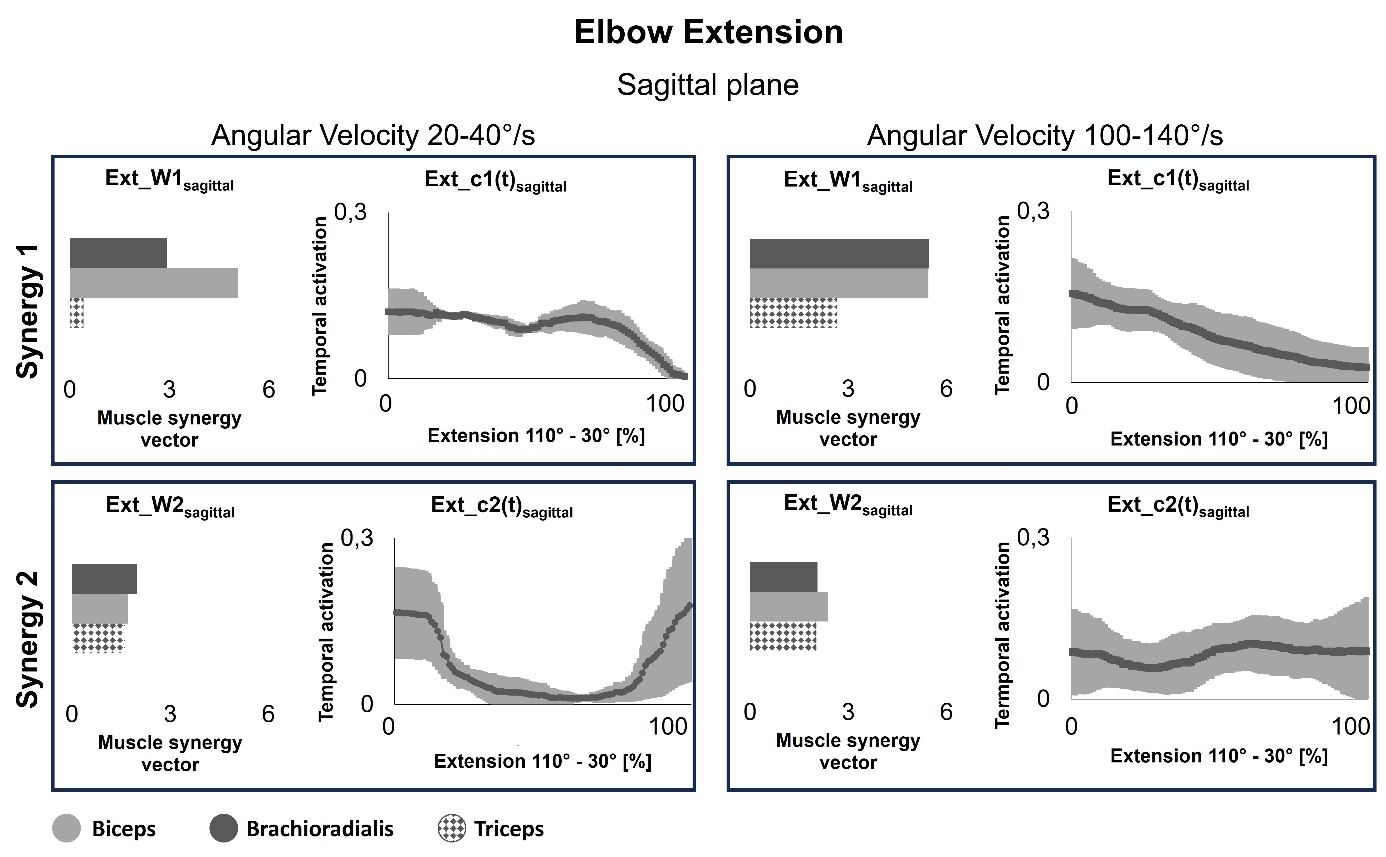
**

**Fig. S1.** Temporal activation coefficients (c(t)) and muscle synergy vectors (W) during elbow extension in the sagittal plane (familiar task). The left column displays the synergies when moving slower (20-40°/s), and the right column shows the synergies when moving fast (100-140°/s).


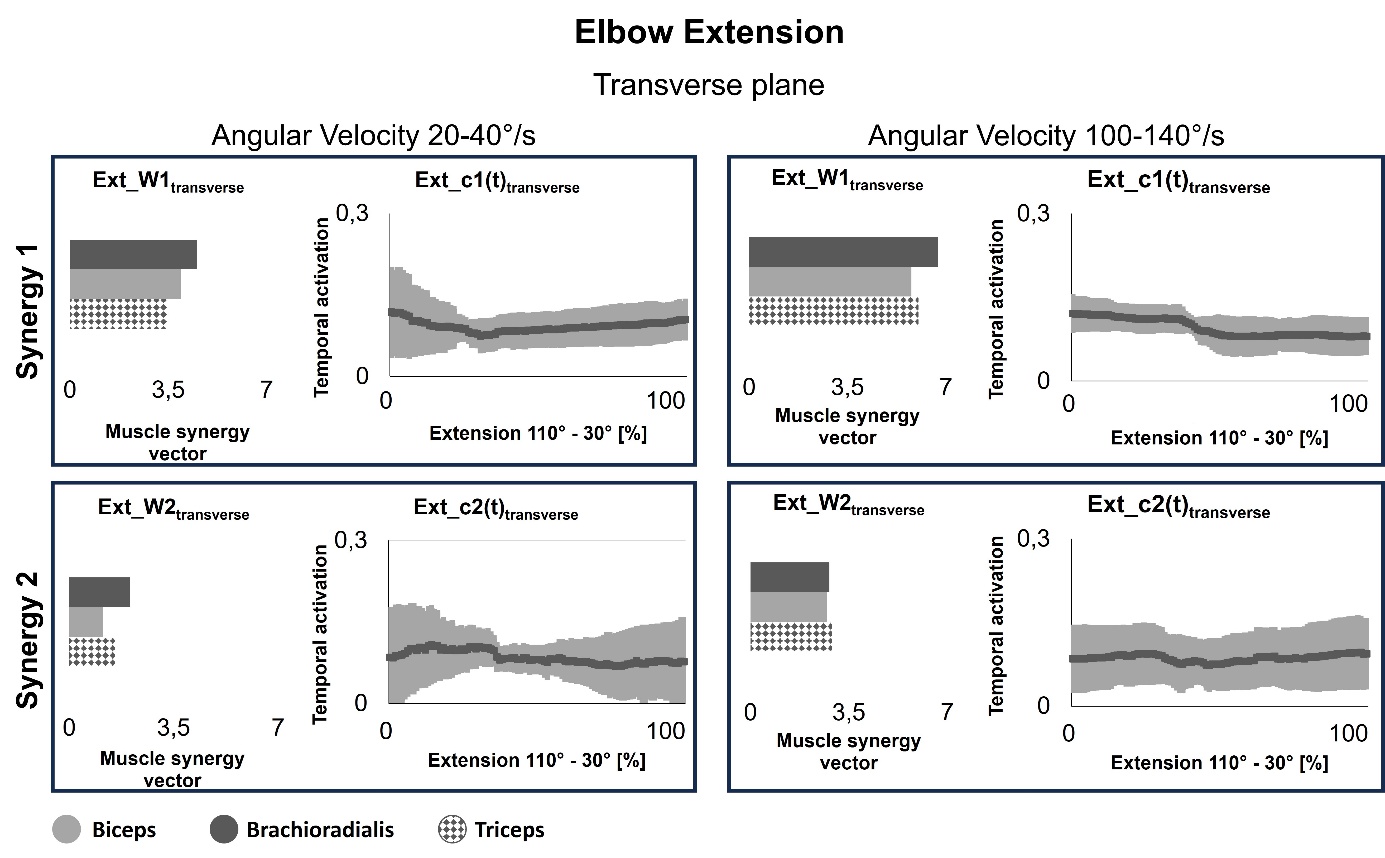


**Fig. S2.** Temporal activation coefficients (c(t)) and muscle synergy vectors (W) during elbow extension in the transverse plane (unfamiliar task). The left column displays the synergies when moving slower (20-40°/s), and the right column shows the synergies when moving fast (100-140°/s).
